# Recruitment of Irgb6 to the membrane is a direct trigger for membrane deformation

**DOI:** 10.3389/fcimb.2022.992198

**Published:** 2022-09-09

**Authors:** Hiroshi Yamada, Tadashi Abe, Hikaru Nagaoka, Eizo Takashima, Ryo Nitta, Masahiro Yamamoto, Kohji Takei

**Affiliations:** ^1^ Department of Neuroscience, Graduate School of Medicine, Dentistry and Pharmaceutical Sciences, Okayama University, Okayama, Japan; ^2^ Division of Malaria Research, Proteo-Science Center, Ehime University, Matsuyama, Japan; ^3^ Division of Structural Medicine and Anatomy, Department of Physiology and Cell Biology, Kobe University Graduate School of Medicine, Kobe, Japan; ^4^ Department of Immunoparasitology, Research Institute for Microbial Diseases, Osaka University, Suita, Japan

**Keywords:** IFN-inducible GTPase, Irgb6, GTPase, membrane, *T. gondii*

## Abstract

Irgb6 is a member of interferon γ-induced immunity related GTPase (IRG), and one of twenty “effector” IRGs, which coordinately attack parasitophorous vacuole membrane (PVM), causing death of intracellular pathogen. Although Irgb6 plays a pivotal role as a pioneer in the process of PVM disruption, the direct effect of Irgb6 on membrane remained to be elucidated. Here, we utilized artificial lipid membranes to reconstitute Irgb6-membrane interaction *in vitro*, and revealed that Irgb6 directly deformed the membranes. Liposomes incubated with recombinant Irgb6 were drastically deformed generating massive tubular protrusions in the absence of guanine nucleotide, or with GMP-PNP. Liposome deformation was abolished by incubating with Irgb6-K275A/R371A, point mutations at membrane targeting residues. The membrane tubules generated by Irgb6 were mostly disappeared by the addition of GTP or GDP, which are caused by detachment of Irgb6 from membrane. Binding of Irgb6 to the membrane, which was reconstituted *in vitro* using lipid monolayer, was stimulated at GTP-bound state. Irgb6 GTPase activity was stimulated by the presence of liposomes more than eightfold. Irgb6 GTPase activity in the absence of membrane was also slightly stimulated, by lowering ionic strength, or by increasing protein concentration, indicating synergistic stimulation of the GTPase activity. These results suggest that membrane targeting of Irgb6 and resulting membrane deformation does not require GTP, but converting into GTP-bound state is crucial for detaching Irgb6 from the membrane, which might coincident with local membrane disruption.

## Introduction

In mammals, infection by a pathogenic microorganism prompts the host to produce interferons (IFNs), cytokines that activate immune systems. Binding of the secreted IFNs to IFN receptors of infected cells activates JACK-STAT signal pathway, which stimulate IFN-stimulated genes(ISGs). So far more than 2,000 ISGs have been identified both in mouse and human. ISGs are highly diverse but integrated in host defense. Among the ISGs, IFN-inducible GTPase superfamily is prominent in that they operate against several pathogenic microorganisms (Kim et al., 2012; MacMicking, 2012; Meunier and Broz, 2016).

Two subfamilies of IFN-inducible GTPases, immunity-related GTPases (IRGs) and guanylate-binding proteins (GBPs), specifically target intracellular vacuolar pathogens and restrict their replication by destroying parasitophorous vacuole (PV), which enfolds parasitizing pathogens (Howard et al., 2011; Yamamoto et al., 2012; Saeij and Frickel, 2017). Infection of *Toxoplasma gondii* (*T. gondii*), an intracellular parasite that causes toxoplasmosis, induces IRGs, which serve for cell-autonomous immunity. IRGs coordinately function in destroying paralytic pathogens in a sequential and hierarchical manner. IRGs are functionally divided into two subfamilies, “regulator” IRGMs and “effector” IRGs. The latter, which include Irga1-10, Irgb1-7, and Irgd, target to and disrupt PVM (Bekpen et al., 2005; Hunn et al., 2008; Khaminets et al., 2010). “Regulator” IRGMs interact with an “effector” IRGs at GDP-bound state, preventing activation of the “effector” IRGs before infection of *T. gondii*. By contrast, GTP-bound “effector” IRGs dimerize and target to PVM (Hunn et al., 2008). Among “effector” IRGs, Irgb6 acts as a pioneer for the recruitment of other “effector” IRGs to PVM (Khaminets et al., 2010; Lee et al., 2019). Thus, mechanisms of cooperation of IRGs in targeting to PVM have been gradually clarified. However, it remains to be solved how Irgb6 attacks PVM.

To analyze the direct effect of Irgb6 on membrane, we tried to reconstitute the interaction of Irgb6 and artificial lipid membrane *in vitro.* Although *in vitro* reconstitution of membrane dynamics is widely used for membrane traffic studies (Takei et al., 2010), and it proved especially useful to clarify functions and dynamics of Dynamin GTPase (Takei et al., 1998; Yoshida et al., 2004; Takeda et al., 2018), it is unknown whether it is applicable for Irgb6. Here we successfully reconstituted the interaction of Irgb6 and artificial lipid membrane, and to analized the direct effect of the interaction.

## Materials and methods

### Recombinant proteins

His-tagged human Irgb6-wild-type (GenBank accession no. NM_001145164: Irgb6-WT) or K275A/R371A mutant (Irgb6- K275A/R371A) were cloned into pEU-E01-MCS expression vector at EcoRI/NotI sites (CellFree Sciences) for the recombinant protein expression. The recombinant proteins were expressed using a wheat germ cell-free expression system (CellFree Sciences), and purified by immobilized-nickel affinity chromatography with Ni Sepharose™ 6 Fast Flow (Cat#17531802, Cytiva). Purification of the proteins were assessed by SDS-PAGE (**Supplementary Figure 1**). Purified proteins were resolved in A buffer (100 mM NaCl, 50 mM Tris-HCl, 500 mM imidazole, pH8.0), and stored at 4°C until use.

### Preparation for liposomes

Liposomes were prepared as previously described (Takeda et al., 2018). Ten % (mol/mol) PI5P:phosphatidylinositol-5-phosphate (Cat#P-5016, Echelon Biosciences), 80% phosphatidylethanolamine (PE; Cat#840022C, Avanti Polar Lipids), 10% cholesterol (Chol; Cat#700000, Avanti Polar Lipids) were mixed in chloroform-methanol mixture (1:3 v/v). Ten % phosphatidylserine (PS; Cat#840032C, Avanti Polar Lipids) or phosphatidylcholine (PC; Cat#840051C, Avanti Polar Lipids) containing lipid mixture were dissolved in chloroform. The lipids were taken in glass tubes, and the solvent was evaporated using slow-flow nitrogen gas to produce a lipid film and then completely dried under vacuum for 1 day. The lipid film was rehydrated by water-saturated nitrogen gas followed by addition of 250 μl of filtered 0.3 M sucrose for 2 h at 37°C. The resultant liposomes were passed through polycarbonate filters with 0.4 μm pore 11 times using Avanti Mini extruder.

### *In vitro* reconstitution of Irgb6-membrane interaction

Liposome solution (0.1 mg/ml) was incubated 1 μM Irgb6 in 100 mM NaCl, 20 mM Tris-HCl, 1 mM DTT, pH 7.5 at 37°C for 15 min or room temperature for 30 min. The samples were absorbed onto a Formvar- and carbon-coated copper grid. To observe the effect of GTP hydrolysis on the liposome deformation, 0.1 mM GTP, 0.1 mM GDP or 0.5 mM GMP-PNP with 1 mM MgCl_2_ were added onto the grid and incubated for 5 min. The grids were negative-stained with 3% uranyl acetate in double deionized H_2_O for 2 min as described (Takei et al., 1998), and observed with a transmission electron microscope (TEM) (H-7650, Hitachi High-Tech Corp.) at a voltage of 120 kV.

### Binding of Irgb6 on lipid-monolayers

Lipid monolayer (PI5P: PE: Chol = 10:80:10 mol/mol) were formed on the surface of a buffer filled in a Teflon block as described (Higgins and McMahon, 2005), and 3 μM Irgb6 in 100 mM NaCl, 20 mM Tris-HCl, 1mM MgCl_2_ 1 mM DTT, pH7.5, with or without 1 mM guanine nucleotides (GTP, GMP-PNP or GDP) was added. A Formvar- and carbon-coated copper grid was placed on the monolayer, incubated for 2 h at room temperature, then the grid was subjected to negative staining and TEM observation as above. Binding of Irgb6 to lipid monolayer, which is visible as uranyl acetate-positive spot at lower magnification images, was quantified as follows. The area corresponding Irgb6 polymers in TEM image (512x512 pixel) taken at ×300 magnification was quantified densitometry using Image J. The 13 images (no nucleotide), 10 images (+ GTP), 11 images (+ GDP) or 13 images (+ GMP-PNP) from independent three to four grids were used for the quantification.

### GTPase assay

To determine the GTPase activity under low ionic- or high ionic-strength conditions, Irgb6-WT resolved in A buffer was dialyzed with 15 mM NaCl, 1 mM DTT, 20 mM HEPES/KOH, pH7.4, or with 100 mM NaCl, 1 mM DTT, 20 mM Tris-HCl, pH7.5, respectively, at 4°C for 16 h. Irgb6 at indicated concentrations were incubated with 2 mM MgCl_2_ and 1 mM GTP at 37°C for 30 min in the presence or absence of 0.1 mg/ml PI5P-containing liposomes. The GTPase reaction was stopped by adding 0.1 M EDTA. GTP hydrolysis was measured using a colorimetric assay to detect inorganic phosphate (Pi) release as previously described (Leonard et al., 2005).

### Statistical analysis

Data were analysed for statistical significance using Kaleida Graph software for Macintosh, version 4.1 (Synergy Software Inc.). Student’s t-tests were used to analyse statistical significance between two groups. All data are displayed as means ± standard error of the means (S.E.M.) with *P* < 0.05 considered statistically significant.

## Results

### Irgb6 deforms PI5P- or PS-containing liposomes in the absence of guanine nucleotides

**Figure 1:**
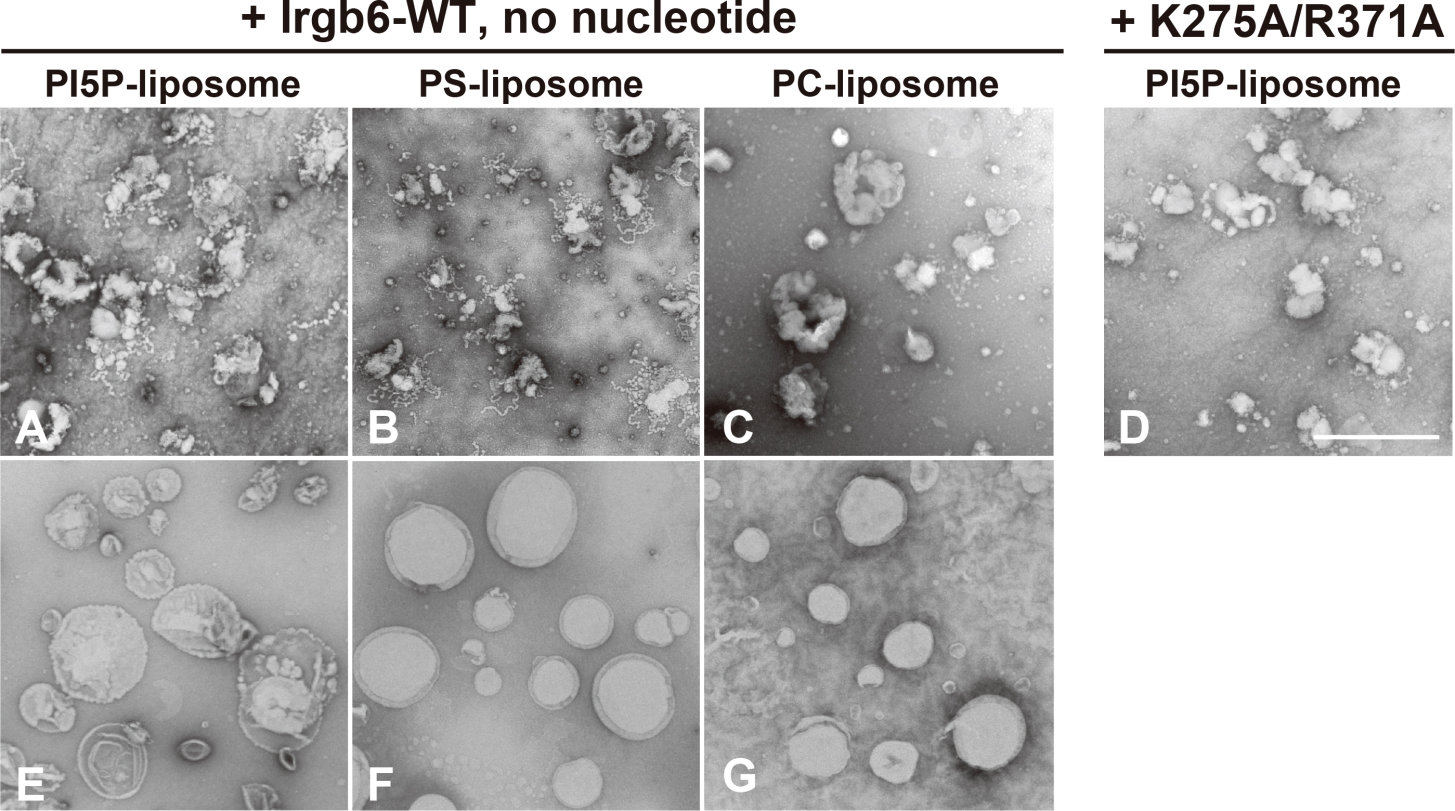
Irgb6 deforms PS or PI5P-containing liposomes. Negative stain EM of 10% PI5P-, PS- or PC-containing liposomes (0.1 mg/ml) incubated with 1 μM Irgb6-WT or K275A/R371A **(A–D)**. Liposome alone were shown **(E–G)**. Note that remarkable membrane deformation of PI5P- or PS-containing liposomes **(A, B)**. Scale bar: 1000 nm **(A–G)**.

To examine direct effect of Irgb6 on lipid membrane, sized unilamellar spherical liposomes containing either 10% of PI5P, PS or PC (**Figures 1E–G**) were incubated with recombinant full length Irgb6 (Irgb6-WT) or mutant Irgb6 (Irgb6-K275A/R371A) in the absence of guanine nucleotides, and observed by TEM. Irgb6-WT prominently deformed PI5P- or PS-containing liposomes, forming multiple bulbar tubules with various length, 20-30 nm in diameter (**Figures 1A, B** **)**. By contrast, deformation and tubulation was much less on PC-containing liposomes (**Figure 1C**). Deformation of PI5P-containing liposomes was drastically reduced by replacing Irgb6-WT with Irgb6-K275A/R371A, a mutant which does not bind to PI5P or PS by dot-blot assay (Lee et al., 2019) (**Figure 1D**). Thus, Irgb6 deformed PI5P- or PS-containing liposomes even in the absence of guanine nucleotide.

### The membrane deformation by Irgb6 is altered by the guanine nucleotide conditions

**Figure 2:**
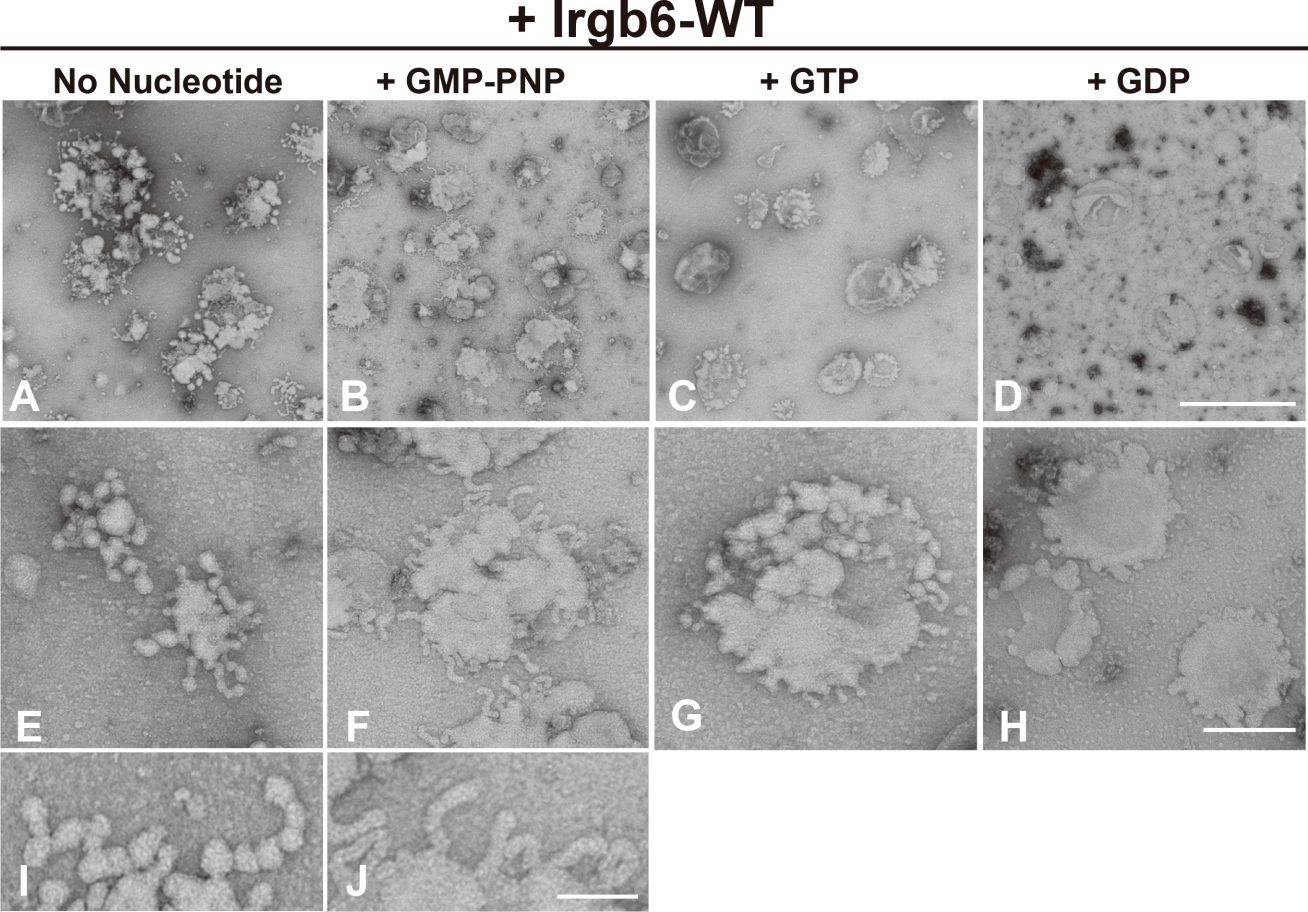
Effect of nucleotide on membrane deformation of Irgb6-WT. Negative stain EM showing deformation of PI5P-containing liposomes by Irgb6-WT under nucleotide conditions as indicated. PI5P-containing liposomes (0.1 mg/ml) incubated 1 μM Irgb6-WT as in **Figure 1** were absorbed on the grids. Then, buffer alone **(A, E, I)**, GMP-PNP at 0.5 mM **(B, F, J)**, GTP at 0.1 mM **(C, G)** or GDP at 0.1 mM **(D, H)** was added and incubated for 5 min as is in **Supplemental Figure 1**. Scale bar: 1000 nm for upper panels **(A–D)**, 200 nm for middle panels **(E–H)**, 75 nm in bottom panels **(I , J)**.

Since Irga6, an “effector” IRG, is recruited to PVM at GTP-bound state (Hunn et al., 2008), we next examined whether the membrane deformation by Irgb6 is changed by its guanine nucleotide conditions. Toward this end, PI5P-containing liposomes deformed by Irgb6 were absorbed on grids, then subjected to various guanine nucleotides (**Supplemental Figure 2**). As described above, membrane deformation by Irgb6 with no nucleotide was mostly tubulo-bulbar, with diameters (26.4 ± 0.5 nm, n = 100) at the dilated portion and (14.0 ± 0.24 nm, n = 80) at the constricted portion (**Figures 2A, E, I** **)**. Addition of GMP-PNP, a unhydrolyzable GTP analogue, which would retain Irgb6 at GTP bound state, resulted in remarkable tubulation. Interestingly, the tubules had a constant diameter, (23.7 ± 0.64 nm, n = 62) (**Figures 2B, F, J** **)**. In contrast, addition of GTP or GDP dramatically lessened the membrane deformation (**Figures 2C, D, G, H** **)**. Thus, membrane deformation by Irgb6 considerably varied depending on guanine nucleotide conditions. This is attributed partly to Irgb6’s membrane affinity changed in guanine nucleotide-dependent manner.

### GTPase activity of Irgb6 is stimulated in the presence of lipid membranes

**Figure 3:**
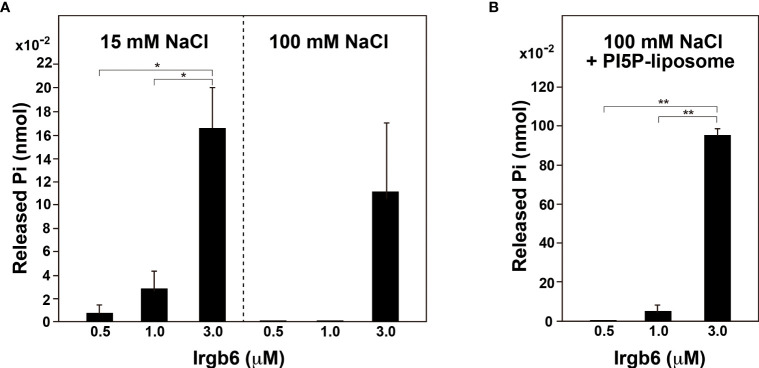
PI5P-containing liposomes stimulate Irgb6 GTPase activity. **(A)** GTPase activity of Irgb6-WT under low (15 mM NaCl) or high (100 mM NaCl) ionic strength conditions. Irgb6-WT at indicated concentrations was incubated in the buffer containing 15 mM or 100 mM NaCl at 37°C for 30 min. Data are means ± S.E.M. of three independent experiments. (*P < 0.05). **(B)** PI5P-containing liposomes stimulate GTPase activity of Irgb6-WT. Irgb6-WT at indicated concentrations were incubated with 0.1 mg/ml PI5P-containing liposomes in high ionic strength condition (100 mM NaCl) at 37°C for 30 min. Data are means ± S.E.M. of three independent experiments. (**P < 0.01).

Dynamin self-assembles under low ionic strength conditions (<50 mM NaCl) (Warnock et al., 1996) or on the lipid membranes (Tuma et al., 1993), and the assembly results in enhancement of dynamin’s GTPase activity. Therefore, we determined the GTPase activity of Irgb6 under low ionic strength conditions or in the presence of liposomes. Under high ionic strength conditions (100 mM NaCl), the GTPase activity of Irgb6-WT was measurable at 3 μM, but undetectable at 0.5 or 1 μM (**Figure 3A** right). By contrast, under low ionic strength condition (15 mM NaCl), the GTPase activity was detectable even at 0.5 μM, and it increased dose-dependent manner (**Figure 3A** left). Irgb6 GTPase activity was even higher in the presence of PI5P-containing liposomes under high ionic strength conditions (**Figure 3B**). The GTPase activity of 3 μM Irgb6 was enhanced by approximately 8.5-fold by the presence of liposomes (compare **Figure 3A** rightmost bar and **Figure 3B** rightmost bar).

### Binding of Irgb6 is changed by the guanine nucleotide conditions

**Figure 4:**
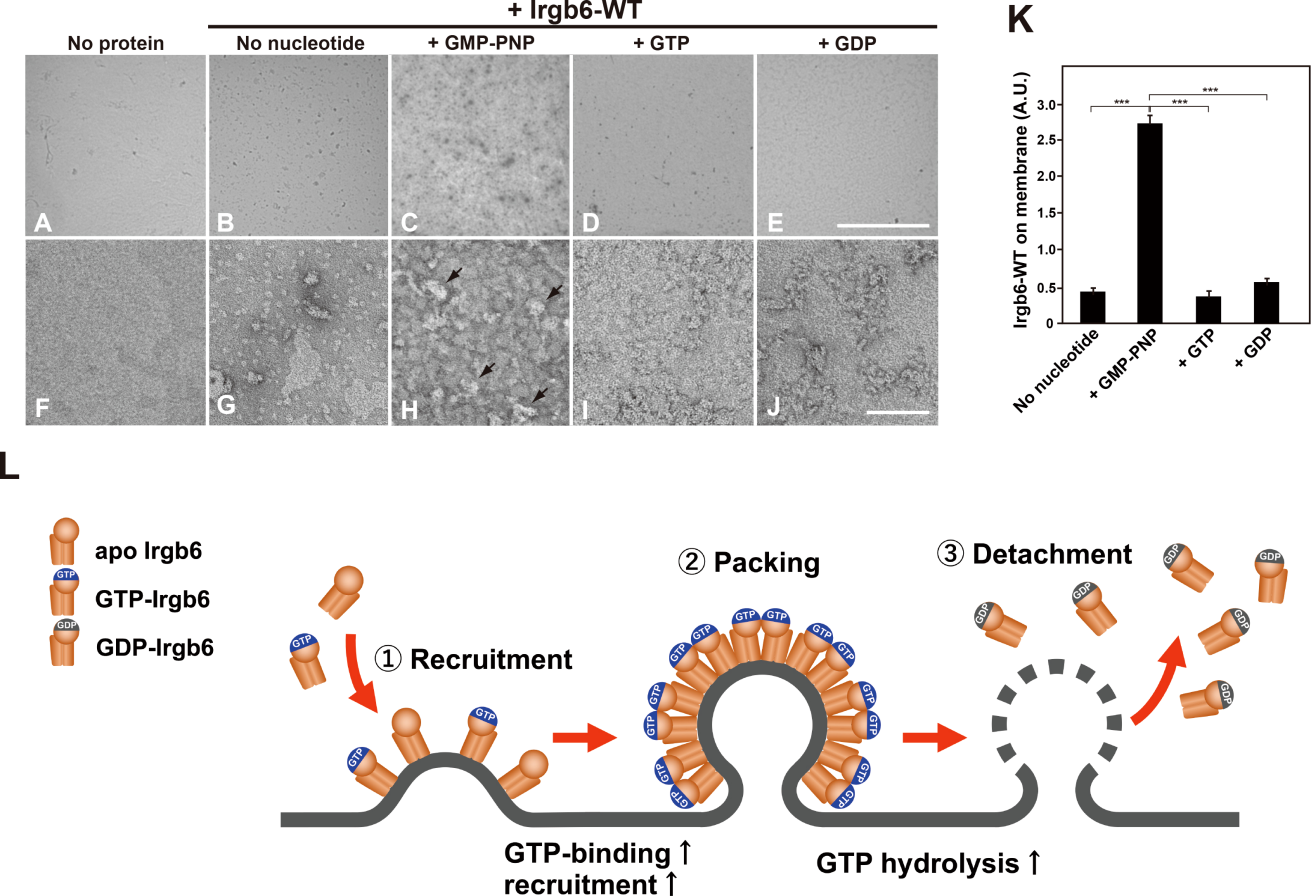
Nucleotide-dependent binding of Irgb6 to PI5P-containing lipid monolayers. **(A–J)** Increased binding of Irgb6 to the lipid monolayers containing PI5P in the presence of GMP-PNP. Three μM Irgb6-WT was absorbed to the lipid monolayers without nucleotide **(B, G)**, or with 1 mM GMP-PNP **(C, H)**, GTP **(D, I)** or GDP **(E, J)** at room temperature for 3 h. TEM image of monolayer without Irgb6 is shown as a negative control **(A, F)**. TEM images taken at 300× (upper panels) or at 30000× (bottom panels) were shown. Note that binding of Irgb6-WT to monolayers was remarkedly increased in the presence of GMP-PNP **(H)**, and some appeared bulged (arrowheads in **H**). Scale bar: 8.6 μm in upper panels **(A–E)**, 100 nm in bottom panels **(F–J)**. **(K)** Quantification of binding of Irgb6-WT to monolayers. Irgb6-WT on the lipid monolayers could be visible in negatively stained TEM images (512 x 512 pixel) taken as in **(A–E)** at low magnification. The area corresponding Irgb6-WT in TEM image was quantified using Image J from three to four independent grids. (***P < 0.0001). **(L)** Model for the molecular machinery of Irgb6 in PVM disruption. Recruitment of Irgb6 to the membrane leads to membrane deformation. The membrane binding is increased at GTP-binding state because of GTP-dependent polymerization of Irgb6. Dense packing of Irgb6 on lipid membrane results in the stimulation of GTPase activity, and upon GTP hydrolysis Irgb6 detaches from the membrane accompanying membrane damage.

Irgb6 differentially deformed liposome membrane depending on the guanine nucleotide conditions (**Figure 2**), suggesting that the binding of Irgb6 to the membrane is varied in guanine nucleotide dependent manner. Therefore, we tried to examine Irgb6-membrane interaction in a more immediate manner. For this purpose, Irgb6 was absorbed on lipid monolayer, and the Irgb6 molecules were observed “en face” by TEM. Irgb6 was recognized as uranyl acetate-positive spot at low magnification (**Figures 4B–E**), and the arrangement was visible at higher magnification (**Figures 4G–J**). Under no nucleotide conditions, majority of Irgb6 appeared as small globular clusters and some formed insular clusters (**Figure 4G** **)**. In the presence of GMP-PNP, Irgb6 globules were larger, condensed (**Figure 4H** **)**, some of which appeared three dimensionally convexed (**Figure 4H** arrows). In the presence of GTP or GDP, Irgb6 formed irregular clusters, but much less crowded compared to these formed in the presence of GMP-PNP (**Figures 4I, J** **)**. Morphometric quantification revealed that the binding of Irgb6 on the membrane was increased more than five-fold compared to other guanine nucleotide conditions (**Figure 4K**).

## Discussion

In this study, we reconstituted Irgb6-membrane interaction using liposomes or lipid monolayer, and demonstrated that Irgb6 prominently deformed liposomes containing PI5P or PS (**Figure 1**). Consistently, Irgb6 binds to PI5P intensely, and to PS, PI3P, and PI4P strongly by protein-lipid overlay assay (Lee et al., 2019). Considering that both PI5P and PS are components of the *T. gondii* PVM, the liposome deformation by Irgb6 is likely to reflect its PV-disrupting function. Irgb6 tubulated liposomes both without nucleotides or at GTP-bound condition (+ GMP-PNP), but the tubules formed with GMP-PNP had constant, and slightly thinner diameters (**Figures 2I, J** **)**. Densely arranged Irgb6 on the membrane at GTP-bound condition (**Figures 4C, H** GMP-PNP) might have caused further deformation.

Irgb6 shares the basic structure with Irga6 and Irgb10, consisting of a GTPase domain, N-terminal domain, and C-terminal domain (Ghosh et al., 2004; Saijo-Hamano et al., 2021; Ha et al., 2021). The N- and C-domains stand side by side and they are composed of parallel or anti-parallel 11 helices, and two long helices located at the center. The long helices bind to the membrane at the bottom, opposite side of GTPase domain (Saijo-Hamano et al., 2021). Irga6 and Irgb10 recognize membrane *via* myristoylated glycine at the N-terminus, which is close to the central pair helices (Ha et al., 2021). Irgb6 lacks such a myristylations site, instead it utilizes two basic residues, K275 and R371, located at the edge of central pair helices. A substitution mutation, Irgb6-K275A/R371A, abolishes lipid binding by protein-lipid overlay assay, and accumulation on PVM of *T. gondii* in infected cells (Lee et al., 2019). By docking simulation, the head groups of PI5P and PS were docked at high affinity on the central helix pair of Irgb6 (Saijo-Hamano et al., 2021). Consistently, membrane deformation by Irgb6 was prominent with PI5P- or PS-containing liposomes, and suppressed with PC-containing liposomes, or with Irgb6-K275A/R371A (**Figure 1**). Thus, Irgb6-membrane interaction, which is dependent on specific lipid species and amino acid residues, and consequent membrane deformation was successfully reconstituted *in vitro*.

IFN-inducible GTPases were formerly grouped in the dynamin GTPase superfamily phylogenetically (Praefcke and McMahon, 2004), and latterly IFN-inducible GTPase superfamily, which includes subfamilies of IRG and GBP, was established (Kim et al., 2012). Compared to other GTPases such as small GTPases, members of dynamin GTPase are relatively higher in molecular weight (∼100 kDa), have a low affinity for GTP (*K*_M_∼10–25 μM), a high basal rate of GTP hydrolysis (*k*_cat_∼8–30×10^−3^ sec^−1^), and an extremely high stimulated rate of GTP hydrolysis (*k*_cat_ 1–5 sec^−1^). Furthermore, the rates for association of GTP (7×10^5^ M^-1^sec^−1^) and for dissociation of GDP (95 sec^−1^) are very rapid. Based on these properties and the intracellular GTP level (≒1 mM), dynamin is thought to exist at nucleotide-free state or GDP bound state only transiently (1–10 ms), and GTP hydrolysis is the rate-limiting step in dynamin’s GTPase cycle  (Sever et al., 2000). Given that Irgb6’s GTPase activity is stimulated by self-assembly or by the presence of membrane as is the case for dynamin (**Figure 3**), the GTPase cycle of Irgb6 might be similar to that of dynamin.

GTPase activities of small G proteins are controlled by GTPase-activating proteins (GAPs) and guanine nucleotide exchange factor (GEFs), whereas those of Dynamin superfamily and IFN-inducible GTPase superfamily are activated by oligomerization/polymerization, and by interaction with lipid membranes (Prakash et al., 2000; Praefcke and McMahon, 2004). Dynamin’s conformation is drastically changed by the presence of membrane. Without membranes, Dynamin is at “closed” conformation having membrane-binding pleckstrin homology domain (PHD) docked near the stalk, a region responsible for dimerization. PHD sticks out to converting dynamin to “open” conformation, which facilitates membrane binding and polymerization (Kong et al., 2018). Furthermore, Dynamin GTPase domain dimerizes across the helical rungs in GTP-dependent manner, which leads to assembly-stimulated GTPase activity and power stroke (Chappie et al., 2010; Chappie et al., 2011). Likewise, GBP1 and Irga6 undergoes GTP-dependent homodimerization, which generates a conformation of GBP1 for efficient catalysis (Ghosh et al., 2006), or accelerates the GTPase activity of Irga6 (Hunn et al., 2008). The recruitment of Irgb6 at GTP-bound state is enhanced approximately 5 – 7.5 fold compared to other guanine nucleotide conditions (**Figures 4A–K**) and the GTPase activity is stimulated eight-fold in the presence of liposomes (**Figure 3**). These properties, together with reported strong homotypic interaction of Irgb6 (Hunn et al., 2008), suggest that Irgb6 also undergoes dimerization/polymerization in GTP-dependent manner.

Taken together, we reconstituted Irgb6-membrane interaction *in vitro*, and demonstrated that Irgb6 directly deformed lipid membrane. The membrane recruitment was controlled in GTPase-dependent manner, and Irgb6 GTPase activity was significantly stimulated by binding to the membrane. Possible molecular machinery of “effector” Irgb6 in PVM disruption is depicted in **Figure 4L**. Currently, it remains unclear how Irgb6 packed on the membrane could disrupt the membrane, although a power stroke of N- and C-domains of is suggested (Saijo-Hamano et al., 2021). Further structural analyses of membrane-bound Irgb6 would solve the open question.

## Data availability statement

The original contributions presented in the study are included in the article/**Supplementary Material**. Further inquiries can be directed to the corresponding authors.

## Author contributions

HY and KT designed the research and wrote the paper. HY, HN, ET, and TA performed the experiments. MY and RN contributed new reagents or analytic tools. All authors read and approved the final manuscript.

## Funding

This work was supported, in part, by grants from the Ministry of Education, Science, Sports, and Culture of Japan (grant numbers 21K19484 to KT, 20K08591 to HY, and 22K06580 to TA), and by the Joint Usage/Research Center for Proteo-Interactome (PRiME), the Proteo-Science Center, Ehime University to HY. This work was supported by the Japan Agency for Medical Research and Development (AMED) (JP22wm0325010 to KT, MY, and RN).

## Acknowledgments

The work was supported by Okayama University Central Research Laboratory.

## Conflict of interest

The authors declare that the research was conducted in the absence of any commercial or financial relationships that could be construed as a potential conflict of interest.

## Publisher’s note

All claims expressed in this article are solely those of the authors and do not necessarily represent those of their affiliated organizations, or those of the publisher, the editors and the reviewers. Any product that may be evaluated in this article, or claim that may be made by its manufacturer, is not guaranteed or endorsed by the publisher.
