# Supplementary figures and images for: Recruitment of Irgb6 to the membrane is a direct trigger for membrane deformation
Source: Front Cell Infect Microbiol. 2022 Sep 9;12:992198. doi: 10.3389/fcimb.2022.992198 (PMC9504060; doi:10.3389/fcimb.2022.992198)

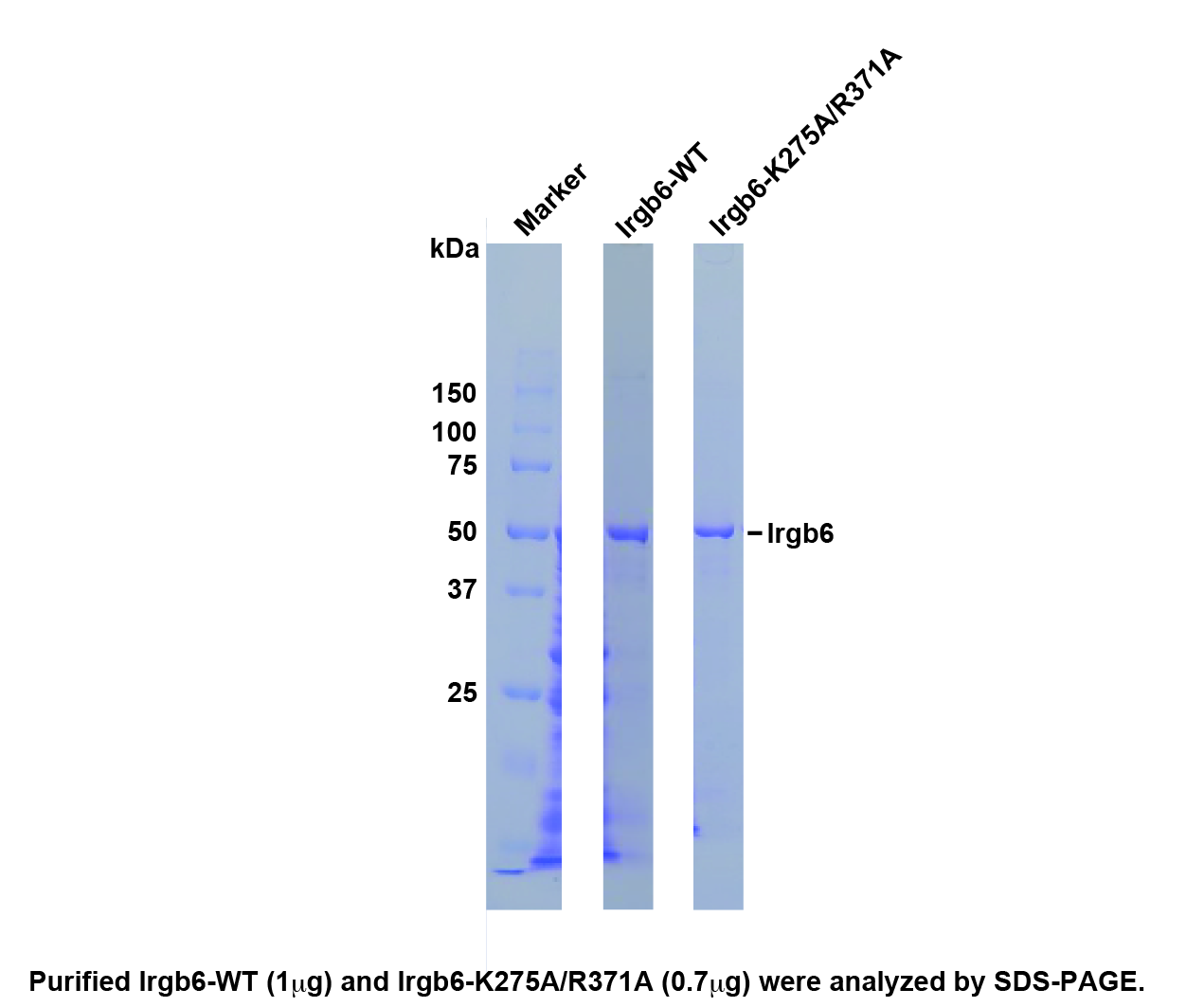

Supplement: Supplementary file 1 [file Image_1.tif]

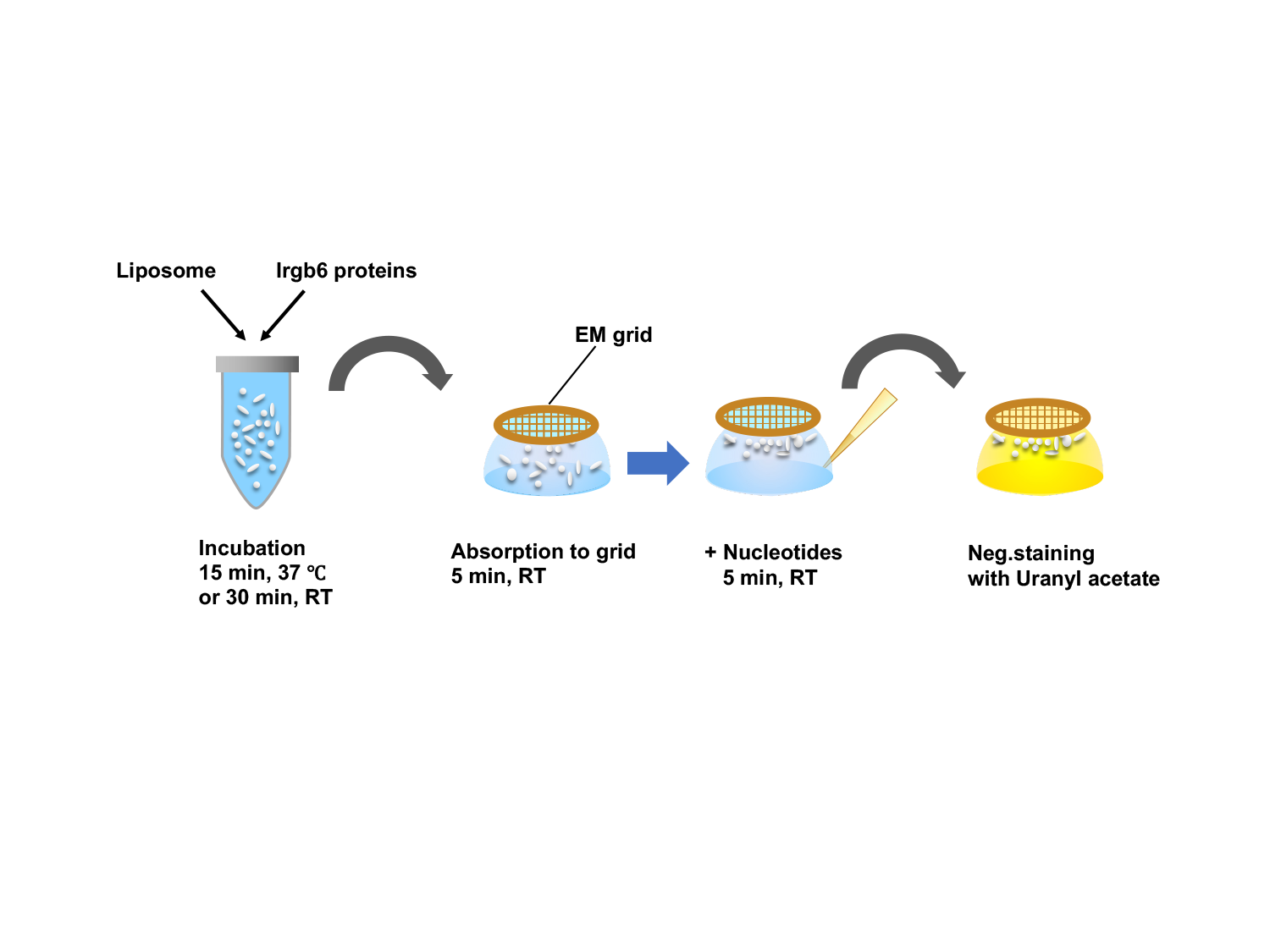

Supplement: Supplementary file 2 [file Image_2.tif]
